# Are clinical measures of foot posture and mobility associated with foot kinematics when walking?

**DOI:** 10.1186/s13047-015-0122-5

**Published:** 2015-11-24

**Authors:** Andrew K. Buldt, George S. Murley, Pazit Levinger, Hylton B. Menz, Christopher J. Nester, Karl B. Landorf

**Affiliations:** Discipline of Podiatry, School of Allied Health, La Trobe University, Bundoora, Melbourne, VIC 3086 Australia; Lower Extremity and Gait Studies Program, School of Allied Health, La Trobe University, Bundoora, Melbourne, VIC 3086 Australia; Institute of Sport, Exercise & Active Living, Victoria University, Footscray, Melbourne, VIC 8001 Australia; School of Health Sciences, University of Salford, Salford, UK

## Abstract

**Background:**

There is uncertainty as to which foot posture measures are the most valid in terms of predicting kinematics of the foot. The aim of this study was to investigate the associations of clinical measures of static foot posture and mobility with foot kinematics during barefoot walking.

**Method:**

Foot posture and mobility were measured in 97 healthy adults (46 males, 51 females; mean age 24.4 ± 6.2 years). Foot posture was assessed using the 6-item Foot Posture Index (FPI), Arch Index (AI), Normalised Navicular Height (NNHt) and Normalised Dorsal Arch Height (DAH). Foot mobility was evaluated using the Foot Mobility Magnitude (FMM) measure. Following this, a five-segment foot model was used to measure tri-planar motion of the rearfoot, midfoot, medial forefoot, lateral forefoot and hallux. Peak and range of motion variables during load acceptance and midstance/propulsion phases of gait were extracted for all relative segment to segment motion calculations. Hierarchical regression analyses were conducted, adjusting for potential confounding variables.

**Results:**

The degree of variance in peak and range of motion kinematic variables that was independently explained by foot posture measures was as follows: FPI 5 to 22 %, NNHt 6 to 20 %, AI 7 to 13 %, DAH 6 to 8 %, and FMM 8 %. The FPI was retained as a significant predictor across the most number of kinematic variables. However, the amount of variance explained by the FPI for individual kinematic variables did not exceed other measures. Overall, static foot posture measures were more strongly associated with kinematic variables than foot mobility measures and explained more variation in peak variables compared to range of motion variables.

**Conclusions:**

Foot posture measures can explain only a small amount of variation in foot kinematics. Static foot posture measures, and in particular the FPI, were more strongly associated with foot kinematics compared with foot mobility measures. These findings suggest that foot kinematics cannot be accurately inferred from clinical observations of foot posture alone.

**Electronic supplementary material:**

The online version of this article (doi:10.1186/s13047-015-0122-5) contains supplementary material, which is available to authorized users.

## Background

Foot posture is characterised by the external shape of the foot and is determined by both the alignment of the bones of the foot and the location, size and mechanical properties of the soft tissues covering them [1]. In standing, foot posture is a measure of the response of the foot to relatively static internal and external forces (the latter applied to the sole of the foot). During dynamic tasks, such as walking, different internal and external forces are applied and these will be task specific. These forces are typically greater than during standing and vary over time.

Measurement of static foot posture and dynamic foot function is considered important as certain structural and functional variations may predispose individuals to injury. For example, planus foot posture has been identified as a risk factor for patellofemoral pain and Achilles tendinopathy [2], while there is some evidence that increased rearfoot peak eversion and eversion velocity is a risk factor for injuries such as patellofemoral pain, Achilles tendinopathy and non-specific lower limb overuse injuries [3]. The mechanism linking static foot posture and dynamic foot function to the development of these conditions has been attributed to altered foot kinematics and loading rates, leading to the development of excessive stress within musculoskeletal tissues [4].

However, the biomechanical literature is inconclusive about the relationships between static foot posture and dynamic foot function [1, 5]. A likely explanation for this is that there are various methods for measuring and classifying static foot posture which limits consistency between studies [1]. Furthermore, many measures provide an incomplete assessment of foot posture. For example, navicular height is the single measure of one bony tuberosity on the medial side of the foot in one cardinal body plane, whereas during gait, motion occurs in many other joints simultaneously and in all planes [6, 7].

We recently conducted a systematic review to better understand the relationship between static foot posture and lower limb kinematics [5]. Some of the studies we included in our review investigated the association between static foot posture and kinematic variables. These studies found that the Foot Posture Index (FPI) can explain between 21 and 85 % of the variation in either peak rearfoot eversion or frontal plane rearfoot range of motion variables. However, the review also identified methodological limitations of the existing literature, including variations in sample selection, exclusion of particular foot postures (e.g. cavus foot), inconsistency in kinematic models used, and selection of a wide range of foot posture measures, many of which have not been well validated.

Of the current static foot posture measures, there are four that have been validated against angular measurements from radiographs, widely considered to be the ‘gold standard’ measurement technique [8–10]. These measures include the FPI [11], the Arch Index (AI) [12], normalised navicular height truncated (NNHt) [11] and dorsal arch height (DAH, also referred to as arch height ratio) [9]. In addition, measures of foot mobility can describe the response of structures to the load applied during the measurement of foot posture, and may, therefore, be more valid in predicting dynamic function than static measurements [13]. The Foot Mobility Magnitude (FMM) has been found to be the most reliable of the current measures of foot mobility [14]. As a result, the use of these measures (i.e. FPI, AI, NNHt, DAH and FMM) may provide useful insights into the motion of the foot during gait.

The aim of this study was to investigate the association between four validated measures of static foot posture and a measure of foot mobility with three dimensional kinematics of the foot during barefoot walking in asymptomatic individuals. Doing so will provide evidence for clinicians who do not typically have access to laboratory-based gait analysis. We hypothesised that measurements of static foot posture or foot mobility that were indicative of an increasing planus foot posture or increased foot mobility would be associated with increased pronatory characteristics, such as greater rearfoot eversion, and increased dynamic joint motion during gait. In completing our analysis the objective was to further evaluate the relationship between foot structure and function.

## Methods

### Participants

Ninety-seven adults (46 males, 51 females) aged 18 to 47 years, who were free of symptoms related to current or recurring lower limb pathology were recruited for this study. Participants were selected from a previous study comparing normal, planus and cavus foot posture groups, hence the sample captured a range of foot postures. The distribution of all foot posture measures in the sample was normal (skewness or kurtosis values were within the range of -2 to 2). Data from only one foot were collected in order to satisfy the assumption of independence for statistical analysis [15]. The single foot to be analysed was selected using a random number generator (Microsoft Excel®, Microsoft Corp, Redmond, Washington, USA). Ethical approval was obtained from the La Trobe University Human Ethics Committee (ID number 11-097) and all participants signed informed consent.

### Static foot posture measurements

The following measurements were undertaken by the same examiner (AKB) and in all instances, the average of three repeated measurements was documented. The static foot posture and foot mobility measures have reported moderate to good intra-rater reliability (ICC range 0.81 – 0.99) [10, 11, 14, 16, 17] and moderate to good inter-rater reliability (ICC range: 0.58 – 0.99) [10, 11, 14, 17, 18]. The static foot posture measures have also displayed concurrent validity against angular measurements obtained from radiographs [8–10], which are regarded as the gold-standard measure of foot posture [13].

#### The 6-item foot posture index (FPI)

The FPI is a multi-planar visual observation tool that comprises the following six assessments: (i) talar head palpation, (ii) curves above and below the lateral malleoli, (iii) frontal plane alignment of the calcaneus, (iv) prominence in the region of the talonavicular joint, (v) height and congruence of the medial longitudinal arch, and (vi) abduction / adduction of the forefoot on the rearfoot [19]. While standing in relaxed bipedal stance, each of these measurements was scored on a 5-point scale between -2 and +2 and then scores for all measurements were summed. Summative scores range between -12 (highly supinated) and +12 (highly pronated). Raw summative scores were converted to Rasch transformed scores to be used as interval data in statistical analysis [20].

#### The arch index (AI)

While standing in relaxed bipedal stance, a static footprint was obtained using Pressurestat® carbon paper (Footlogic Inc, South Salem, NY, USA). A foot axis was drawn from the centre of the posterior heel to the tip of the second toe. The footprint (excluding the toes) was divided into three regions of equal length, and the area of each region calculated using a tablet computer (Toshiba Tecra A9, Toshiba Corp, Minato, Tokyo, Japan) and diagramming software (Microsoft Visio, Microsoft Corp, Redmond, Washington, USA). The AI was calculated as a ratio of area of the middle region of the footprint to the area of the complete footprint excluding the toes [12].

#### Normalised navicular height truncated (NNHt)

While standing in relaxed bipedal stance, the most medial prominence of the navicular was marked and the height (mm) from the supporting surface was measured [11]. The measurement was normalised to truncated foot length, which was obtained by measuring the length of the foot between the posterior heel and the most medial aspect of the first metatarsophalangeal joint. NNHt was calculated by dividing navicular height by truncated foot length.

#### Dorsal arch height (DAH)

**Fig. 1:**
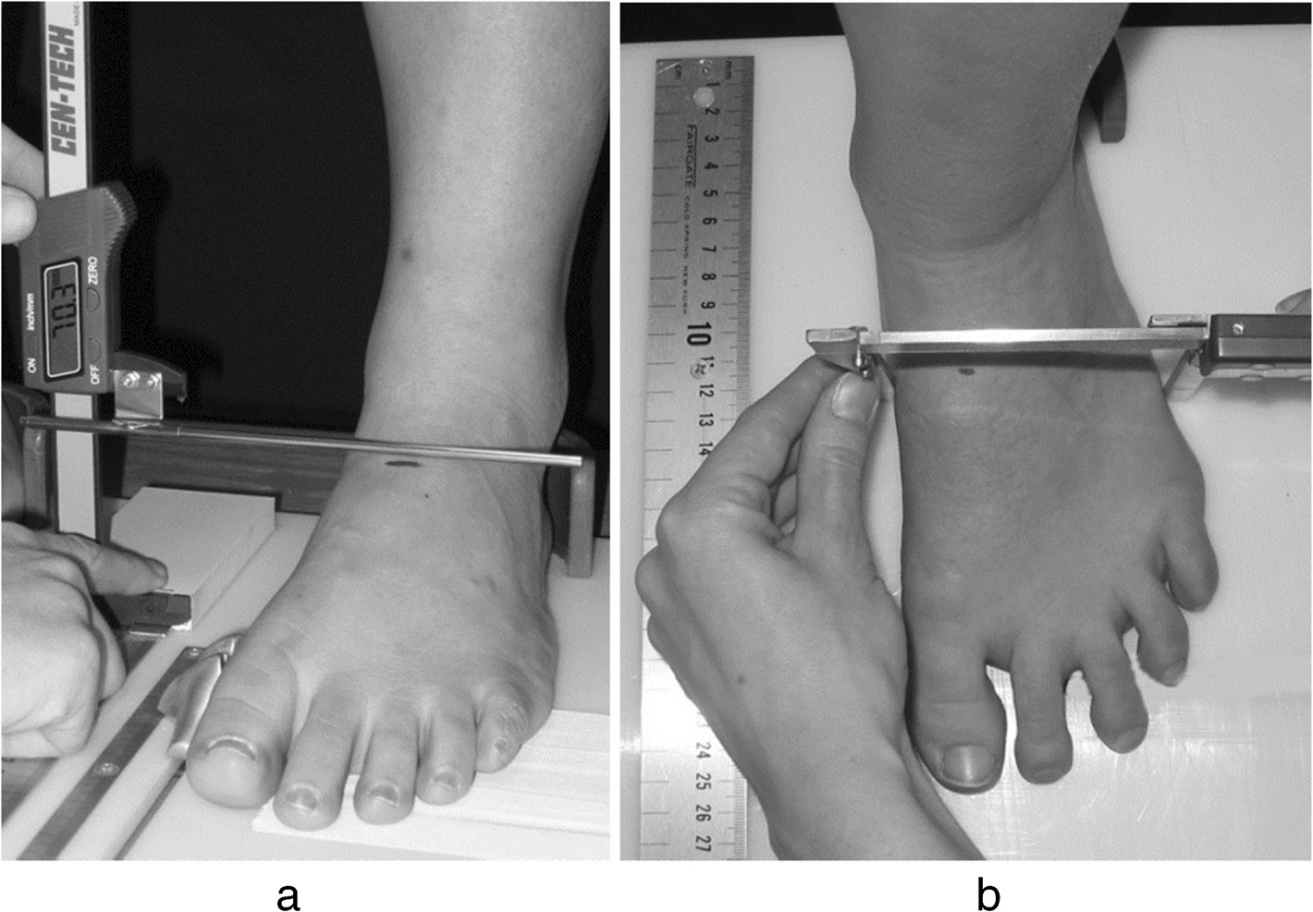
Procedure for the measurement of midfoot height (**a**) and midfoot width (**b**) to calculate foot mobility magnitude. Platform was also used for the measurement of DAH. Image first appeared in Journal of Foot and Ankle Research (2009) [14]

Participants were asked to assume relaxed bipedal stance with the tested foot on a measurement platform and the heel positioned in a fixed heel cup. The dorsum of the foot at 50 % of foot length was marked, and foot length was measured as the distance between the most posterior aspect of the calcaneus and the most distal aspect of the longest toe. A vertical digital caliper measured the height of the dorsal marking from the supporting surface. DAH was calculated by dividing the arch height with truncated foot length obtained during the calculation of NNHt [9]. The procedure for the measurement DAH is illustrated in Fig. 1.

### Foot mobility measurement

#### Foot mobility magnitude (FMM)

A measurement platform that was specifically designed for undertaking this measurement was used [14]. Participants were asked to assume relaxed bipedal stance with both heels in heel cups positioned 15 cm apart. The 50 % dorsal foot marking was used and the dorsal arch height measurement obtained using the same procedure as the DAH measurement. The weightbearing midfoot width at 50 % of foot length was measured by using a digital caliper to measure the distance between the medial and lateral side of the foot. The procedure for the measurement of weightbearing midfoot height and width is illustrated in Fig. 1.

To assess non-weightbearing midfoot height and width, the participant was asked to sit on the edge of a table with both legs hanging perpendicular to the floor in a relaxed position. Non-weightbearing arch height was measured by slowly moving a portable platform vertically until it made slight contact with the plantar surface of the foot. The participant was asked to give feedback to the examiner when they could feel the platform in contact with the medial forefoot, lateral forefoot and heel simultaneously, while not forcibly pushing any contact point into dorsiflexion. The height of the arch at 50 % foot length was measured using a digital caliper attached to the portable platform. To measure non-weightbearing midfoot width, the distance between the medial and lateral aspect of the foot at 50 % of foot length was measured. All measurements were undertaken three times and the average of the three measurements presented in centimetres for calculation [15].

To determine the FMM, the difference between the non-weightbearing and weightbearing measures of the midfoot height and width was calculated. The following formula was used to calculate the FMM:

$$ FMM=\sqrt{}{\left( difference\kern0.5em  midfoot\kern0.5em  height\right)}^2+{\left( difference\kern0.5em  midfoot\kern0.5em  width\right)}^2 $$

### Anthropometric, spatiotemporal and clinical covariates

In order to control for the influence of other measurements that may influence foot kinematics, a range of anthropometric, spatiotemporal and clinical measurements were collected. These included height, weight, body mass index (BMI), foot length (using the procedure outlined for obtaining DAH) and truncated foot length (using the procedure outlined for obtaining NNHt). Spatiotemporal measurements were obtained from kinematic data and included stride length, step length and walking speed obtained from the heel contact timing data. Clinical joint flexibility measures were also calculated. In all instances, the average of three repeated measurements was documented by the same investigator. The measurements are were follows:

#### Active weight bearing dorsiflexion of the ankle joint (straight leg lunge test)

The participant stood with both hands on a wall and positioned the foot that was to be tested as far as possible behind the contralateral foot while ensuring the knee of the tested foot was fully extended. The centre of the heel and second toe was positioned on a line that was perpendicular to the wall. The participant was instructed to lean forward until maximum stretch was sensed, while keeping the knee extended and the heel on the ground. The angle of the tested leg at the midpoint of the anterior tibial border between the tibial tuberosity and the anterior joint line was measured using an inclinometer [21].

#### Passive non weight bearing inversion and eversion of the ankle/subtalar joint

Prior to measurement the participant performed four full active ankle inversion and eversion cycles. The participant was seated on the edge of a plinth with the lower leg over the edge of the bed unsupported and the ankle in a relaxed position. Angles were measured with a universal goniometer at the midpoint between the malleoli on the anterior aspect of the ankle, with the arms of the goniometer placed on the midline of the anterior aspect of the leg (using the tibial crest as a reference point) and the longitudinal midline of the dorsal surface of the second metatarsal. The investigator applied an inversion force to the calcaneus until end range of motion (ROM) was reached and then repeated for eversion [22, 23]. Total ankle motion, incorporating both inversion and eversion was also recorded.

#### Passive non-weight bearing dorsiflexion of the first metatarsophalangeal joint

The midline of the medial aspect of the hallux and the medial aspect of the 1^st^ metatarsal were bisected longitudinally. A dorsiflexion force was applied to the proximal phalanx of the hallux until end ROM was reached. The angle between the markings was measured with a universal goniometer [24].

### Kinematic instrumentation

A three-dimensional motion analysis system comprising ten cameras (eight MX2 and two MX8, Vicon motion systems Ltd, Oxford, England) with a sampling frequency of 100 Hz was used to capture foot kinematics. Gait cycle events were detected using two force plates with a sampling frequency of 1000 Hz (Kistler, type 9865B, Winterthur, Switzerland and AMTI, OR6, USA).

### Kinematic procedure

**Fig. 2:**
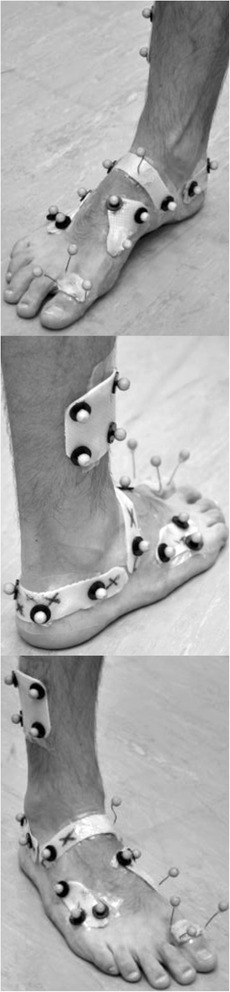
Marker placement and orientation of the foot for static reference trial

Foot kinematics were recorded using a five segment foot model and marker set [25, 26]. Nine millimetre retro-reflective markers were mounted on thermoplastic plates. The plates were heated to a malleable state and then placed on the foot of the participants while weightbearing. The plates then cooled to adopt a stiffer state that was shaped according to the dimensions of the participants’ foot [6]. A representation of the marker set is shown in Fig. 2. A static calibration trial was recorded with the participant asked to stand in relaxed bipedal stance position. Local coordinate frames were defined for each segment with the longitudinal axis of the calcaneus aligned parallel to previously defined anterio-posterior axis of the global system. The relaxed standing reference position was defined as 0 degrees for calculation of segment to segment motion in the sagittal, transverse and frontal planes. Familiarisation walking trials were then undertaken until the participant was comfortable with the testing instrumentation. Participants were asked to walk at a self-selected pace on a 12 m walkway. A minimum of five acceptable trials (whereby the tested foot landed within the first force plate and the contralateral foot landed in the second force plate) were recorded. Walking speed, stride length and step length were calculated using force plate data, and trials were processed if they were within 10 % of the walking speed of the last familiarisation trial.

### Data reduction

Each acceptable trial was reconstructed and processed using Vicon Nexus Software (Vicon motion systems Ltd, Oxford, England). This process comprises the following steps: identify the trajectory of each marker, label each marker and identify trajectories, fill gaps and filter (using Woltring method). All data was normalised via interpolation to 0-100 % of the entire gait cycle and was exported to Microsoft Excel (Microsoft Corporation, Redmond, WA, USA) for data extraction. Relevant data was extracted from five acceptable trials. The following relative segment to segment motion calculation were analysed for the sagittal, transverse and frontal planes.

(i) rearfoot (calcaneus) relative to the leg;
(ii) midfoot (navicular, cuboid) relative to the rearfoot;
(iii) medial forefoot (cuneiforms and metatarsals 1, 2 and 3) relative to the midfoot;
(iv) lateral forefoot (metatarsals 4 and 5) relative to the midfoot;
(v) hallux relative to the medial forefoot.

Peak angle of dorsiflexion/plantarflexion, abduction/adduction and inversion/eversion were extracted between 0 and 70 % of the gait cycle. This range was used as it captures kinematic variables during stance and early swing phase. Such a technique was adopted to characterise foot kinematics during stance phase, and to ensure consistency of data extraction between participants by preventing the inclusion of peak variables during swing phase that may influence results. In addition, range of motion (ROM), determined by the peak to peak segmental excursions was extracted in the time periods spanning 0 to 20 % and 20 to 70 % of the gait cycle. These periods were analysed as they relate to initial loading, midstance, propulsion and early swing phase periods of gait respectively.

### Statistical analysis

Multiple regression analyses were undertaken to determine how static foot posture measures and the FMM measure are associated with peak and ROM kinematic variables. However, due to the high number of kinematic variables, an *a-priori* decision was made to only analyse kinematic variables that exhibited at least a moderate linear relationship (Pearson’s *r* > 0.3) [27] with static foot posture or the FMM measure. To account for the influence of covariates, all anthropometric and spatiotemporal variables that exhibited significant relationships (*p* < 0.05) with kinematic variables were included in the corresponding regression model.

Hierarchical regression models were then undertaken. Covariates were entered in block 1. To reduce the influence of collinearity, the variance inflation factor was assessed. When this value exceeded 5, the related covariate was identified and the covariate that had the lowest correlation with the kinematic variable was eliminated from the regression model. The static foot posture or foot mobility measure was entered into block 2. Adjusted *R*^2^ values and adjusted *R*^2^ change variables were calculated for all regression models. All statistical tests were calculated using SPSS version 21 for Windows (IBM Corporation, NY, USA).

## Results

**Fig. 3:**
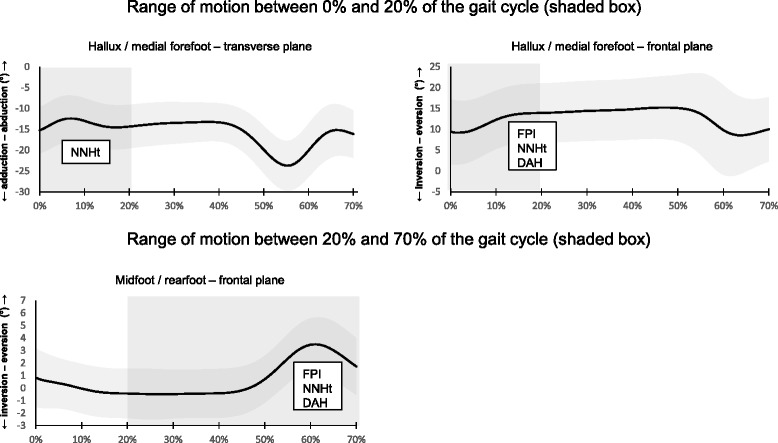
Average ensemble and retained foot posture measures for range of motion kinematic variables included in regression analyses. Standard deviation is indicated by shaded band

**Fig. 4:**
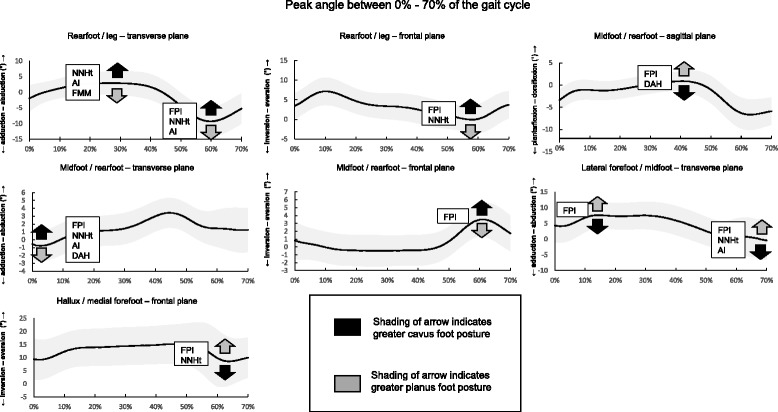
Average ensemble and retained foot posture measures for peak kinematic variables included in regression analyses with indication of the direction of relationship with foot posture measure. Standard deviation is indicated by shaded band

Participant characteristics, foot posture, anthropometric and spatio-temporal measurements are presented in Table 1. Hierarchical regression models are presented in Table 2 and graphical representations of the kinematic variables in each regression model and the direction of the association with foot posture measures are illustrated in Figs. 3 and 4. Bivariate correlations between all foot posture and foot mobility measures and both ROM and peak angle variables appear in Additional files 1, 2 and 3. Only associations that were analysed in regression models are described. To aid interpretation, the terms ‘increasing cavus’ or ‘increasing planus’ foot posture is used throughout the Results and Discussion to illustrate the direction of the association between foot posture measures and kinematic variables. It is not a description of foot posture classification categories. For completeness, the results relating to covariates are also reported.

**Table 1:** Participant characteristics, values are mean ± SD

Gender (*n*)	Male: 46, Female: 51
Age (years)	24.4 (6.2)
Static foot posture / foot mobility measures	
FPI (Rasch transformed)	2.5 (3.1)
AI	0.2 (0.1)
NHHt	0.2 (0.1)
DAH	0.4 (0.1)
FMM	1.60 (0.4)
Anthropometric measurements	
Height (cm)	171.5 (10.4)
Weight (kg)	70.1 (14.4)
BMI (kg/m^2^)	23.7 (3.0)
Foot length (cm)	251.8 (20.3)
Truncated foot length (cm)	177.3 (14.5)
Spatiotemporal measurements	
Stride length (m)	1.4 (0.1)
Step length (m)	0.7 (0.1)
Walking speed (m/s)	1.3 (0.1)
Additional measurements	
Active weightbearing dorsiflexion of the ankle (°)	41.7 (9.1)
Passive non weightbearing inversion of ankle / subtalar joint (°)	36.6 (9.2)
Passive non weightbearing eversion of ankle / subtalar joint (°)	13.9 (6.1)
Passive non weightbearing total sagittal plane ROM of ankle / subtalar joint (°)	50.3 (12.2)
Passive non weightbearing 1^st^metatarsophalangeal joint dorsiflexion (°)	73.2 (15.9)

*FPI* Foot Posture Index, *AI* Arch index, *NNHt* Normalised navicular height truncated, *DAH* Dorsal arch height, *FMM* Foot mobility magnitude, *BMI* Body mass index

**Table 2:** Regression models of the kinematic variables

ROM kinematic variables. 0-20 % of the gait cycle							
Kinematic variable – plane of motion	ROM Variable	Variables in block 1	Block 1 adjusted *R* ^2^	Foot posture measure added	Block 2 adjusted *R* ^2^	Change in *R* ^2^	*P* value
Hallux-medial forefoot – transverse	Abduction	-	-	NNHt	0.110	-	0.001
Hallux-medial forefoot – frontal	Eversion	Step length	0.089	FPI	0.196	0.113	<0.001
		Truncated foot length		NNHt	0.238	0.153	<0.001
		1^st^ MTPJ dorsiflexion		DAH	0.161	0.079	0.003
ROM kinematic variables. 20-70 % of the gait cycle							
Midfoot-rearfoot – frontal	Eversion	Step length	0.092	FPI	0.170	0.084	0.002
		Height		NNHt	0.178	0.092	0.001
		1^st^ MTPJ dorsiflexion		DAH	0.148	0.063	0.009
Peak kinematic variables							
Kinematic variable – plane of motion	Peak variable	Variables in block 1	Block 1 adjusted *R* ^2^	Foot posture measure added	Block 2 adjusted *R* ^2^	Change in *R* ^2^	*P* value
Rearfoot / leg – transverse plane	Abduction	-		NNHt	0.097	-	0.001
				AI	0.087	-	0.002
				FMM	0.080	-	0.003
Rearfoot / leg – transverse plane	Adduction	Foot length	0.039	FPI	0.263	0.222	<0.001
		Truncated foot length		NNHt	0.238	0.199	<0.001
		Ankle Inversion		AI	0.164	0.129	<0.001
		Ankle ROM					
Rearfoot / leg – frontal plane	Inversion	Step length	0.116	FPI	0.205	0.093	0.001
		Height		NNHt	0.198	0.086	0.002
		Weight					
		Ankle Inversion					
		Ankle ROM					
Midfoot / rearfoot – sagittal plane	Dorsiflexion	1st MTPJ dorsiflexion	0.053	FPI	0.114	0.070	0.007
				DAH	0.121	0.076	0.005
Midfoot / rearfoot – transverse plane	Adduction	Height	0.207	FPI	0.311	0.108	<0.001
		Weight		NNHt	0.322	0.188	<0.001
		Truncated foot length		AI	0.278	0.076	0.002
				DAH	0.286	0.084	0.001
Midfoot / rearfoot – frontal plane	Eversion	1st MTPJ dorsiflexion	0.040	FPI	0.139	0.106	0.001
		Ankle inversion					
Lat forefoot / midfoot – transverse plane	Abduction	Stride length	0.073	FPI	0.123	0.057	0.014
		Weight					
		Ankle inversion					
		Ankle ROM					
Lat forefoot / midfoot – transverse plane	Adduction	Stride length	0.143	FPI	0.241	0.099	0.001
		Height		NNHt	0.198	0.060	0.009
		Weight		AI	0.207	0.068	0.005
		Lunge					
		Ankle eversion					
		Ankle ROM					
Hallux / medial forefoot – frontal plane	Inversion	Foot length	0.100	FPI	0.180	0.085	0.002
		Truncated foot length		NNHt	0.099	0.063	0.011
		1st MTPJ dorsiflexion					
		Ankle inversion					

*FPI* Foot Posture Index, *NNHt* Normalised navicular height truncated, *AI* Arch index, *DAH* Dorsal arch height, *FMM* Foot mobility magnitude

### Association between foot posture measures and kinematic variables

DAH explained 7.9 % of the variation in abduction ROM of the hallux between 0 and 20 % of the gait cycle and 6.3 % of the variation in eversion ROM of the midfoot between 20 and 70 % of the gait cycle. This measure also explained between 7.6 and 8.4 % of the variation in peak midfoot dorsiflexion and adduction.

The AI explained between 6.8 and 12.9 % of the variation in peak abduction and adduction of the rearfoot, peak adduction of the midfoot and peak adduction of the lateral forefoot.

NNHt explained between 11.0 and 15.3 % of the variation in abduction and eversion ROM between 0 and 20 % of the gait cycle and 9.2 % of the variation in midfoot eversion between 20 and 70 % of the gait cycle. This measure also explained between 6.0 and 19.9 % of the variation in peak abduction, adduction and inversion of the rearfoot, peak adduction of the midfoot, peak adduction of the lateral forefoot and peak inversion of the hallux.

The FPI explained 11.3 % of the variation in eversion ROM of the hallux between 0 and 20 % of the gait cycle and 8.4 % of the variation in midfoot eversion between 20 and 70 % of the gait cycle. This measure also explained between 5.7 and 22.2 % of the variation in peak adduction and inversion of the rearfoot, peak dorsiflexion, adduction and eversion of the midfoot, peak abduction and adduction of the lateral forefoot and peak inversion of the hallux.

The FMM measure explained 8 % of the variation in peak rearfoot abduction.

### Direction and timing of relationships between foot posture measures and kinematic variables

Cavus foot posture was used as the reference posture to describe the direction of the associations between foot posture measures and kinematic variables. In all instances, the opposite relationship with greater planus foot postures is implied (Fig. 4). The direction of associations was the same for all foot posture measures.

For *ROM kinematic variables*, foot posture measures that were indicative of an increasing cavus foot posture were associated with increased abduction and eversion ROM of the hallux between 0 and 20 % of the gait cycle and increased eversion ROM of the midfoot between 20 and 70 % of the gait cycle.

For *peak kinematic variables*, foot posture measures indicative of increasing cavus foot posture and a decreased FMM measure (i.e. less midfoot mobility) were associated with increased peak rearfoot abduction during midstance, reduced rearfoot adduction during propulsion and reduced inversion of the rearfoot during propulsion. Greater cavus foot posture was also associated with increased peak midfoot adduction during initial contact, reduced dorsiflexion during midstance and increased eversion during propulsion. Finally, increasing cavus foot posture was associated with decreased peak lateral forefoot abduction during initial contact, increased adduction during early swing, and increased peak eversion of the hallux during early swing phase.

### Associations of anthropometric, spatiotemporal and clinical covariates with kinematic variables

Anthropometric and spatiotemporal variables explained 8.9 % of the variation in abduction ROM of the hallux between 0 and 20 % of the gait cycle, and 9.2 % of variation in eversion ROM of the midfoot between 20 and 70 % of the gait cycle (Table 2). Furthermore, these measures explained between 3.9 and 20.0 % of the variation in peak kinematic variables of the rearfoot, midfoot, lateral forefoot and hallux.

## Discussion

The aim of this study was to investigate the association between clinical measures of static foot posture or mobility and foot kinematics during barefoot walking in asymptomatic individuals. We found several statistically significant, independent associations between measures of static foot posture or foot mobility and kinematic variables. However, in no case was more than 22 % of the variation in either peak angle or ROM variables explained. The practical interpretation of these results is that, when carried out on a wide range of foot postures, the use of clinical measures of foot posture or foot mobility can explain only a small amount of variation in dynamic kinematic behaviour of the foot when walking. We also hypothesised an association between increasing planus foot posture and dynamic pronatory characteristics. The results indicated little evidence of this association.

Compared to other static foot posture measures and the FMM measure, the FPI was retained as a significant predictor across a greater number of kinematic variables than all other measures. This suggests that the FPI, a measure consisting of multiple observations in all three planes of motion, may allow for a more comprehensive association with foot kinematics than measures that consist of a single, uniplanar observation (which is the case for NNHt and or DAH). However, the amount of variability that was explained by the FPI was ultimately similar to other foot posture measures as demonstrated by the small range of *R*^2^ change values between foot posture measures. For example, the kinematic variable that displayed the largest range of adjusted *R*^2^ change values was peak midfoot adduction. In this instance, the *R*^2^ change values ranged from 0.076 (for AI) to 0.188 (for NNHt), a range of only 0.112. This indicates that the foot posture measures identified as significant predictors in regression analyses display a similar ability to predict individual kinematic variables.

The FMM measure displayed weaker associations with all kinematic variables when compared to static foot posture measures. In particular, we did not expect that there would be a lack of association of the FMM with midfoot ROM measures, since the FMM measure focuses on changes in midfoot position with the application of load during weightbearing stance. This may be due to markedly different external loading patterns applied to the foot during gait compared to standing and the different contribution of internal forces such as muscle activity. Compared to individuals with less foot mobility, those displaying greater foot mobility scores may require greater force to be applied by muscles that counteract external pronatory forces applied to the midfoot. This hypothesis is supported by studies that have found differences between normal and pes planus foot posture groups in muscle activity and tendon thickness of muscles that control midfoot motion [28, 29]. To further test this hypothesis, future studies should investigate the relationship between FMM and EMG in muscles such as tibialis posterior and peroneus brevis and longus.

Foot posture measures were more strongly associated with foot peak angle kinematic variables compared to ROM kinematic variables. For example, a more planus foot posture was associated with increased peak *adduction* and *inversion* of the rearfoot (i.e. a more supinated position) during propulsion, yet a similar association was not found with the degree of transverse or frontal plane motion during this time period. While the marginally stronger relationship with foot positioning may suggest variations in muscle activity in response to external forces, it may also be explained by overall differences in foot position captured by the relaxed standing position used to capture the static calibration trial. The apparently counter-intuitive finding of greater peak rearfoot *inversion* and *adduction* during propulsion was found in a previous study that used comparable methods to ours in relation to reference positioning, finding greater peak rearfoot inversion in a planus foot group compared to a normal group during the propulsion [30]. The same study also reported vastly different results in peak rearfoot frontal plane angle when using an examiner determined ‘neutral’ reference position [30], thus highlighting the importance of the reference position when interpreting foot kinematic research. The ‘neutral’ reference position approach was not used in our study due to its questionable reliability [30] and significant doubts with regard to whether this position is reached during gait [31].

The findings related to peak frontal plane angle of the rearfoot in our study were inconsistent with the only other study by Chuter [31] that investigated the association between the FPI and peak rearfoot kinematics, which reported a strong *positive* association between the FPI measures indicative of increasing planus foot posture and peak rearfoot eversion (Pearson’s *r* = 0.92). In contrast, our study reported a weak negative association between FPI and peak rearfoot eversion (*r* = -0.26) and it was not included in the regression analyses. However, peak eversion occurred at differing times between the two studies. In our study, peak eversion occurred during initial contact, while in Chuter’s study [31], it occurred during pre-swing. Our study also included participants with cavus foot posture that are more likely to record larger peak eversion angle values using a relaxed weight bearing reference position [32], whereas Chuter investigated only normal and planus postures.

While the associations between static foot posture measures and ROM kinematic variables were comparatively weak, there were findings that may be significant from a functional perspective. For example, during the propulsive phase of gait (between 50 and 70 % of the gait cycle), a foot with a greater cavus posture score displayed greater eversion motion of the midfoot on the rearfoot, while a more planus foot posture score displayed less motion. This increased compliance of the midfoot in the frontal plane is similar to that found in the simulated weightbearing kinematic evaluation of cadaver specimens by Okita and colleagues [33]. Such findings question longstanding theories that the midfoot and forefoot can withstand increased loading during propulsion by increasing ‘rigidity’ of the foot [34]. Instead, these findings suggest that the midfoot undergoes co-ordinated eversion motion during the propulsion that may vary according to static foot posture.

Strengths of this study include the relatively large sample size, the inclusion of participants with a range of foot postures, and the use of a kinematic foot model that addresses the rigid body assumption of kinematic analysis. However, the findings of this study should also be interpreted in the context of three key limitations. Firstly, healthy young individuals were recruited for this study, so our findings may not be generaliseable to older or symptomatic populations, or those with marked foot deformity not related to foot posture. However, given the minimal differences in kinematics between symptomatic and asymptomatic groups reported by some investigators [35, 36], it is unlikely that the fundamental nature of the relationship between static measures of foot posture and mobility in standing, and the kinematics of the foot in walking would differ greatly if symptomatic participants were included. Secondly, while we adjusted for several anthropometric, spatiotemporal and clinical covariates in our regression models, there are likely to be other factors we did not measure that may influence these associations. Thirdly, the method for defining local coordinate systems during a static trial is independent of variations in foot placement that may occur during gait.

## Conclusion

The static measures of foot posture and foot mobility investigated in this study could explain only a small amount of variance in foot kinematics during walking amongst asymptomatic participants with a range of foot postures. Of the variance that was explained, foot posture measures displayed stronger associations with peak angle kinematic variables compared to ROM kinematic variables, and the FPI displayed the strongest association with kinematic variables compared with other foot posture and foot mobility measures. These findings suggest that foot kinematics cannot be accurately inferred solely from observations of foot posture that are commonly undertaken in clinical practice.

## Additional files

Additional file 1:
**Bivariate correlations (Pearson’s r) between foot posture measures and ROM between 0-20 % of gait.** (DOCX 14 kb) Additional file 2:
**Bivariate correlations (Pearson’s r) between foot posture measures and ROM between 20 %-70 % of gait.** (DOCX 14 kb) Additional file 3:
**Bivariate correlations (Pearson’s r) between foot posture measures and peak kinematic variables.** (DOCX 17 kb)
