# Supplementary material for: Are clinical measures of foot posture and mobility associated with foot kinematics when walking?
Source: J Foot Ankle Res. 2015 Nov 24;8:63. doi: 10.1186/s13047-015-0122-5 (PMC4657281; doi:10.1186/s13047-015-0122-5)
Supplement: Additional file 1: — Bivariate correlations (Pearson’s r) between foot posture measures and ROM between 0-20 % of gait. (DOCX 14 kb) [file 13047_2015_122_MOESM1_ESM.docx]

**Additional file 1.** Bivariate correlations (Pearson’s r) between foot posture measures and ROM between 0-20% of gait.

| **Segmental relationship** | **Plane of motion** | **FPI** | | **NNHt** | | **AI** | | **DAH** | | **FMM** | |
| --- | --- | --- | --- | --- | --- | --- | --- | --- | --- | --- | --- |
|  |  | **r** | ***P* value** | **r** | ***P* value** | **r** | ***P* value** | **r** | ***P* value** | **r** | ***P* value** |
| Rearfoot relative to  tibia | Sagittal | -0.058 | 0.571 | 0.076 | 0.462 | -0.037 | 0.720 | -0.027 | 0.796 | -0.127 | 0.216 |
|  | Transverse | 0.218 | 0.032 | 0.230 | 0.023 | 0.225 | 0.026 | 0.229 | 0.024 | 0.222 | 0.029 |
|  | Frontal | 0.159 | 0.120 | -0.129 | 0.207 | 0.060 | 0.558 | -0.113 | 0.271 | 0.078 | 0.450 |
| Midfoot relative to  rearfoot | Sagittal | 0.147 | 0.151 | -0.155 | 0.129 | 0.135 | 0.187 | -0.026 | 0.789 | 0.135 | 0.188 |
|  | Transverse | 0.241 | 0.047 | -0.197 | 0.053 | 0.140 | 0.173 | -0.160 | 0.117 | 0.044 | 0.672 |
|  | Frontal | 0.124 | 0.227 | -0.049 | 0.631 | 0.086 | 0.402 | -0.008 | 0.934 | 0.023 | 0.825 |
| Medial forefoot  relative to  midfoot | Sagittal | -0.109 | 0.287 | 0.203 | 0.046 | -0.065 | 0.528 | 0.091 | 0.374 | -0.003 | 0.974 |
|  | Transverse | -0.165 | 0.107 | 0.228 | 0.025 | -0.121 | 0.240 | 0.181 | 0.076 | -0.101 | 0.356 |
|  | Frontal | 0.084 | 0.413 | -0.077 | 0.451 | 0.083 | 0.416 | 0.098 | 0.338 | -0.129 | 0.209 |
| Lateral forefoot  relative to  midfoot | Sagittal | 0.056 | 0.586 | -0.039 | 0.707 | 0.003 | 0.976 | -0.081 | 0.429 | -0.061 | 0.550 |
|  | Transverse | -0.168 | 0.099 | 0.178 | 0.081 | -0.135 | 0.188 | 0.078 | 0.445 | -0.062 | 0.545 |
|  | Frontal | 0.018 | 0.861 | 0.036 | 0.725 | -0.050 | 0.625 | 0.031 | 0.765 | 0.009 | 0.930 |
| Hallux relative  to medial  forefoot | Sagittal | -0.119 | 0.247 | 0.114 | 0.266 | -0.086 | 0.401 | 0.081 | 0.429 | -0.046 | 0.657 |
|  | Transverse | -0.298 | 0.003 | 0.345 | 0.001 | -0.201 | 0.049 | 0.231 | 0.023 | 0.224 | 0.027 |
|  | Frontal | -0.424 | 0.000 | 0.479 | 0.000 | -0.257 | 0.011 | 0.344 | 0.001 | -0.148 | 0.148 |

FPI – Foot Posture Index, NNHt – Normalised navicular height truncated, AI – Arch index, DAH – Dorsal arch height, FMM – Foot mobility magnitude
