# Supplementary material for: Are clinical measures of foot posture and mobility associated with foot kinematics when walking?
Source: J Foot Ankle Res. 2015 Nov 24;8:63. doi: 10.1186/s13047-015-0122-5 (PMC4657281; doi:10.1186/s13047-015-0122-5)
Supplement: Additional file 2: — Bivariate correlations (Pearson’s r) between foot posture measures and ROM between 20 %-70 % of gait. (DOCX 14 kb) [file 13047_2015_122_MOESM2_ESM.docx]

**Additional file 2.** Bivariate correlations (Pearson’s r) between foot posture measures and ROM between 20%-70% of gait.

| **Segmental relationship** | **Plane of motion** | **FPI** | | **NNHt** | | **AI** | | **DAH** | | **FMM** | |
| --- | --- | --- | --- | --- | --- | --- | --- | --- | --- | --- | --- |
|  |  | **r** | ***P* value** | **r** | ***P* value** | **r** | ***P* value** | **r** | ***P* value** | **r** | ***P* value** |
| Rearfoot relative to  tibia | Sagittal | -0.086 | 0.402 | 0.179 | 0.079 | -0.092 | 0.371 | 0.133 | 0.193 | -0.159 | 0.120 |
|  | Transverse | 0.253 | 0.012 | -0.177 | 0.083 | 0.131 | 0.099 | -0.091 | 0.374 | 0.044 | 0.668 |
|  | Frontal | 0.006 | 0.953 | 0.069 | 0.500 | -0.056 | 0.585 | -0.069 | 0.499 | -0.140 | 0.171 |
| Midfoot relative to  rearfoot | Sagittal | 0.086 | 0.403 | -0.144 | 0.160 | 0.072 | 0.480 | -0.178 | 0.081 | 0.027 | 0.791 |
|  | Transverse | 0.034 | 0.741 | -0.050 | 0.624 | 0.031 | 0.765 | -0.029 | 0.779 | -0.076 | 0.457 |
|  | Frontal | -0.378 | 0.000 | 0.385 | 0.000 | -0.278 | 0.006 | 0.309 | 0.002 | 0.156 | 0.128 |
| Medial forefoot  relative to  midfoot | Sagittal | 0.175 | 0.087 | -0.163 | 0.111 | 0.164 | 0.107 | -0.200 | 0.050 | 0.281 | 0.005 |
|  | Transverse | 0.004 | 0.972 | 0.005 | 0.959 | -0.047 | 0.649 | 0.064 | 0.536 | -0.196 | 0.054 |
|  | Frontal | 0.152 | 0.137 | -0.098 | 0.342 | 0.146 | 0.154 | -0.079 | 0.444 | 0.048 | 0.640 |
| Lateral forefoot  relative to  midfoot | Sagittal | -0.010 | 0.919 | -0.003 | 0.977 | -0.157 | 0.123 | -0.042 | 0.681 | -0.048 | 0.644 |
|  | Transverse | -0.172 | 0.092 | 0.147 | 0.151 | -0.263 | 0.009 | 0.090 | 0.383 | -0.050 | 0.627 |
|  | Frontal | -0.151 | 0.139 | 0.139 | 0.174 | -0.266 | 0.009 | 0.046 | 0.658 | -0.071 | 0.489 |
| Hallux relative  to medial  forefoot | Sagittal | -0.057 | 0.581 | 0.096 | 0.393 | -0.088 | 0.394 | 0.010 | 0.921 | 0.005 | 0.959 |
|  | Transverse | -0.222 | 0.030 | 0.234 | 0.021 | -0.293 | 0.004 | 0.193 | 0.060 | 0.223 | 0.029 |
|  | Frontal | -0.285 | 0.005 | 0.298 | 0.003 | -0.254 | 0.013 | 0.217 | 0.034 | -0.160 | 0.120 |

FPI – Foot Posture Index, NNHt – Normalised navicular height truncated, AI – Arch index, DAH – Dorsal arch height, FMM – Foot mobility magnitude
