# Supplementary material for: Are clinical measures of foot posture and mobility associated with foot kinematics when walking?
Source: J Foot Ankle Res. 2015 Nov 24;8:63. doi: 10.1186/s13047-015-0122-5 (PMC4657281; doi:10.1186/s13047-015-0122-5)
Supplement: Additional file 3: — Bivariate correlations (Pearson’s r) between foot posture measures and peak kinematic variables. (DOCX 17 kb) [file 13047_2015_122_MOESM3_ESM.docx]

**Additional file 3.** Bivariate correlations (Pearson’s r) between foot posture measures and peak kinematic variables.

| **Segmental relationship** | **Plane of motion** | **Peak variable** | **FPI** | | **NNHt** | | **AI** | | **DAH** | | **FMM** | |
| --- | --- | --- | --- | --- | --- | --- | --- | --- | --- | --- | --- | --- |
|  |  |  | **r** | ***P* value** | **r** | ***P* value** | **r** | ***P* value** | **r** | ***P* value** | **r** | ***P* value** |
| Rearfoot  relative  to leg | Sagittal | Plantarflexion 1 | -0.015 | 0.885 | -0.015 | 0.887 | 0.015 | 0.888 | -0.066 | 0.519 | 0.051 | 0.617 |
|  |  | Dorsiflexion | -0.142 | 0.164 | 0.143 | 0.162 | -0.126 | 0.219 | 0.121 | 0.234 | -0.117 | 0.253 |
|  |  | Plantarflexion 2 | 0.002 | 0.985 | -0.080 | 0.436 | 0.015 | 0.885 | -0.050 | 0.628 | 0.076 | 0.462 |
|  | Transverse | Abduction | -0.298 | 0.003 | 0.326 | 0.001 | -0.310 | 0.002 | 0.253 | 0.012 | -0.300 | 0.003 |
|  |  | Adduction | -0.467 | 0.000 | 0.425 | 0.000 | -0.379 | 0.000 | 0.294 | 0.003 | -0.289 | 0.004 |
|  | Frontal | Eversion | -0.265 | 0.009 | 0.286 | 0.005 | -0.287 | 0.004 | 0.209 | 0.040 | 0.234 | 0.021 |
|  |  | Inversion | -0.386 | 0.000 | 0.357 | 0.000 | -0.275 | 0.006 | 0.280 | 0.005 | 0.135 | 0.187 |
| Midfoot  relative  to rearfoot | Sagittal | Dorsiflexion | 0.314 | 0.002 | -0.285 | 0.005 | 0.266 | 0.008 | -0.323 | 0.001 | 0.050 | 0.627 |
|  |  | Plantarflexion | 0.120 | 0.243 | -0.065 | 0.526 | 0.124 | 0.226 | -0.072 | 0.483 | 0.052 | 0.616 |
|  | Transverse | Abduction | -0.488 | 0.000 | 0.487 | 0.000 | -0.370 | 0.000 | 0.435 | 0.000 | 0.162 | 0.113 |
|  |  | Adduction | -0.150 | 0.144 | 0.201 | 0.049 | -0.159 | 0.121 | 0.244 | 0.016 | -0.050 | 0.624 |
|  | Frontal | Eversion | -0.073 | 0.475 | 0.016 | 0.875 | 0.022 | 0.833 | 0.018 | 0.858 | 0.011 | 0.912 |
|  |  | Inversion | -0.324 | 0.001 | 0.280 | 0.005 | -0.159 | 0.120 | 0.192 | 0.059 | -0.087 | 0.397 |
| Medial  FF relative  to midfoot | Sagittal | Dorsiflexion | -0.009 | 0.930 | 0.015 | 0.887 | -0.018 | 0.860 | -0.032 | 0.759 | 0.095 | 0.355 |
|  |  | Plantarflexion | -0.139 | 0.177 | 0.119 | 0.247 | -0.123 | 0.232 | 0.123 | 0.234 | -0.145 | 0.159 |
|  | Transverse | Abduction | -0.057 | 0.582 | 0.051 | 0.624 | -0.103 | 0.317 | 0.104 | 0.315 | 0.066 | 0.523 |
|  |  | Adduction | -0.021 | 0.841 | 0.007 | 0.942 | -0.071 | 0.490 | 0.022 | 0.831 | 0.046 | 0.658 |
|  | Frontal | Inversion | -0.015 | 0.844 | 0.043 | 0.675 | 0.070 | 0.498 | 0.014 | 0.895 | 0.183 | 0.074 |
|  |  | Eversion | -0.075 | 0.486 | 0.073 | 0.480 | 0.009 | 0.928 | 0.037 | 0.722 | 0.165 | 0.109 |
| Lateral  FF relative  to midfoot | Sagittal | Dorsiflexioj | -0.089 | 0.387 | 0.091 | 0.375 | -0.001 | 0.992 | 0.119 | 0.245 | 0.102 | 0.322 |
|  |  | Plantarflexion | -0.124 | 0.226 | 0.117 | 0.255 | 0.007 | 0.947 | 0.178 | 0.081 | 0.155 | 0.130 |
|  | Transverse | Abduction | 0.318 | 0.001 | 0.237 | 0.019 | 0.175 | 0.087 | -0.124 | 0.225 | -0.059 | 0.565 |
|  |  | Adduction | 0.432 | 0.000 | -0.340 | 0.001 | 0.337 | 0.001 | -0.184 | 0.071 | -0.021 | 0.839 |
|  | Frontal | Eversion | -0.134 | 0.185 | 0.079 | 0.439 | 0.043 | 0.679 | -0.021 | 0.839 | 0.128 | 0.210 |
|  |  | Inversion | -0.094 | 0.398 | 0.026 | 0.804 | 0.093 | 0.365 | -0.034 | 0.740 | 0.120 | 0.240 |
| Hallux  relative  to medial FF | Sagittal | Plantarflexion | -0.042 | 0.682 | 0.001 | 0.996 | -0.011 | 0.917 | 0.002 | 0.988 | 0.114 | 0.270 |
|  |  | Dorsiflexion | -0.091 | 0.379 | 0.103 | 0.320 | -0.091 | 0.379 | 0.038 | 0.715 | 0.055 | 0.591 |
|  | Transverse | Abduction | -0.218 | 0.033 | 0.174 | 0.090 | -0.230 | 0.024 | 0.104 | 0.316 | -0.241 | 0.018 |
|  |  | Adduction | -0.007 | 0.950 | 0.039 | 0.705 | 0.005 | 0.964 | -0.085 | 0.408 | -0.043 | 0.676 |
|  | Frontal | Eversion | 0.009 | 0.388 | 0.119 | 0.249 | 0.033 | 0.746 | -0.150 | 0.145 | 0.045 | 0.662 |
|  |  | Inversion | 0.304 | 0.003 | 0.320 | 0.002 | 0.217 | 0.034 | -0.300 | 0.003 | 0.163 | 0.113 |

NNHt – Normalised navicular height truncated, FPI – Foot Posture Index, DAH – Dorsal arch height, AI – Arch index, FMM – Foot mobility magnitude
